# A Dynamic and Complex Early Inflammatory Response in Blood and Cerebrospinal Fluid of Severe Traumatic Brain Injury Patients: A Dual Platform Analysis

**DOI:** 10.1007/s10753-026-02549-9

**Published:** 2026-06-26

**Authors:** Oskar W. Persson, Karsten Ruscher, Erik Uvelius, Niklas Marklund

**Affiliations:** 1 Department of Neurosurgery, Skåne University Hospital, Lund, S-22184 Sweden; 2 Department of Clinical Sciences, Neurosurgery, Lund University, Lund, S-22184 Sweden

**Keywords:** Traumatic brain injury, Inflammation, Electrochemiluminescence, Proximity extension assay, Cytokines, Cerebrospinal fluid

## Abstract

Severe traumatic brain injury (TBI) is associated with high mortality and long-term disability. Inflammation is central to TBI pathophysiology, yet early dynamics of inflammatory mediators in blood and cerebrospinal fluid (CSF) remain incompletely understood, and commonly used assay platforms have rarely been directly compared. We aimed to characterize the inflammatory response in blood and CSF during the first week after severe TBI and to assess agreement between electrochemiluminescence (ECL) and proximity extension assay (PEA). In this prospective observational study, adults with severe TBI (*n* = 21) were recruited. Plasma and CSF samples were collected at two time points: days 1–3 and days 4–8. Orthopedic patients with minor extremity fractures (*n* = 11) served as controls. Inflammatory mediator levels were quantified using ECL (11 mediators) and PEA (45 mediators). Group differences, temporal changes, and inter-platform agreement were analyzed. In plasma, 13 mediators were increased and 3 decreased, while in CSF, 19 were increased and 3 decreased during the first week post-injury. Key mediators (IL-6, IL-8, IL-10) were consistently elevated in both compartments. When comparing analytic methods, ECL and PEA showed strong cross-platform correlations for IL-8 and IL-10 in both plasma and CSF, whereas IL-13 showed weak, non-significant correlation. However, limited interchangeability between platforms and platform-related differences across compartments and time points were also observed. Severe TBI is associated with marked, temporal and compartmentalized inflammatory response during first post-injury, particularly in CSF. Comparison of ECL and PEA showed limited interchangeability, underscoring the importance of platform awareness and interpretation in TBI biomarker studies. Clinical trial number: Not applicable.

**Supplementary Information:**

The online version contains supplementary material available at 10.1007/s10753-026-02549-9.

## Introduction

Traumatic brain injury (TBI) is a global health problem and a leading cause of death and disability in young adults [1]. An immediate, primary injury to brain tissue, caused by direct mechanical forces, occurs at the moment of impact. Later, complex secondary injury mechanisms develop over hours, weeks, or even years after the initial trauma and markedly exacerbate the primary injury [2]. These secondary injuries are due to complications such as ischemia, hemorrhages, excitotoxicity and cerebral edema. In addition, an inflammatory response is initiated early post-injury and often persists long after the TBI. This chronic neuroinflammation is linked to white matter atrophy and neurodegeneration [3, 4]. One way to measure inflammation is by quantifying biomarkers of inflammatory mediators, including cytokines and chemokines, small proteins that regulate inflammatory signaling and cell trafficking [4]. The importance of early inflammation has been debated, as both detrimental and pre-regenerative mechanisms may be activated. Recently, the early inflammatory response was found to be related to the injury pattern and may be used to identify patients at risk of a poor neurological outcome [5, 6]. Moreover, most reports have analyzed post-injury blood samples and evaluated a limited number of inflammatory factors. While it is known that both pro- and anti-inflammatory biomarkers/inflammatory mediators fluctuate during the acute and sub-acute phase of TBI, few studies have explored the temporal pattern of these changes in both blood and cerebrospinal fluid (CSF) [7]. Furthermore, the relative contributions of systemic (blood) *versus* central (in cerebrospinal fluid; CSF) inflammation remain poorly understood in severe TBI, and activation of endogenous retroviruses after CNS injury may trigger type I interferon pathways that may be detected in blood and CSF [8]. 

Electrochemiluminescence (ECL) and Proximity Extension Assay (PEA) are two technologies commonly used to measure inflammatory biomarkers. The well-established ECL method is utilized in various commercial platforms, enabling the simultaneous quantification of multiple analytes with high sensitivity and specificity [9]. It has been widely applied in research on inflammatory and neurodegenerative disorders [10]. In contrast, PEA is a newer, highly multiplexed platform that employs antibody pairs labeled with complementary DNA oligonucleotides to detect proteins with exceptional specificity and a broad dynamic range [11]. There are claims of a higher sensitivity and a lower threshold for detection with PEA, although the technique has rarely been compared directly to ECL methods [5, 12]. To our knowledge, no previous study has directly compared these two technologies in severe TBI, particularly with parallel analyses of both plasma and CSF compartments.

In the present study, we used and compared two biomarker detection techniques, ECL and PEA, in the measurement of inflammatory mediators in plasma and CSF, from patients with severe TBI at two time points during the first week post-injury. We hypothesized there would be a robust, yet complex inflammatory response with an altered pattern over time and with differences between the two compartments.

## Methods

### Study Design

This study was a prospective observational study that conveniently recruited patients with severe TBI between January 2022 – June 2024 at Skåne University Hospital in Lund, Sweden. Patients were recruited from the Neurointensive Care Unit (NICU) within 72 h of injury. All severe TBI patients aged between 18 and 80 years, with an expected NICU stay of one week, were eligible for inclusion. Severe TBI was defined as a Glasgow Coma Scale (GCS) score ≤ 8, using the last pre-intubation assessment recorded at the primary admitting hospital. The presence of any pupillary abnormalities was also noted. A list of exclusion criteria is provided in Suppl. Table 1.

A control group comprised of orthopedic patients, aged 18–75 years old and without a history of neurodegenerative disease, undergoing spinal anesthesia for minor fractures in the lower extremities, was also recruited. Blood and CSF were collected at time of spinal anesthesia.

### Clinical Data Collection and Outcome Assessment

Basic demographic and clinical data, including age, sex, cause of injury, and length of NICU stay, were collected. Surviving TBI patients were routinely contacted by phone 9–12 months post-injury to assess long-term outcome although this was not further analyzed. Functional outcome using structured follow-up interviews using the Extended Glasgow Outcome Scale (GOS-E) were all conducted by a trained study nurse.

### Collection of Samples

Blood and CSF samples were collected simultaneously from the TBI patients twice: first, within three days post-injury (early time point) and again four to eight days post-injury (late time point). Blood samples from the TBI patients were drawn from a peripheral arterial catheter and allocated into EDTA-coated tubes. CSF was collected from patients who had received an external ventricular drain (EVD) for intracranial pressure (ICP) monitoring, by aspirating and discarding a small initial volume and then sampling freshly obtained CSF from the system. All samples were centrifuged at 2000 rpm (relative centrifugal force, RCF, of 644 g) for 10 min at 4 °C to separate plasma and cellular components. Supernatants were allocated to 500 µl tubes and stored at -80 °C within one hour of sampling until analysis.

Samples from the control group were collected prior to the onset of orthopedic surgery at the time of spinal anesthesia. CSF was collected using a sterile 2 ml tube during lumbar puncture before injection of spinal anesthetics. Venous blood samples were collected from the patient at the same time as CSF was collected and allocated into EDTA-coated tubes. These samples were prepared and stored in the same way as samples from TBI patients.

All samples were handled by two study group participants (SM and OP) using the same approach and protocol as described in previous paragraphs.

## Biomarker Analysis

### Proximity Extension Assay

For PEA analysis, samples were sent on dry ice to SVAR Life Science AB, Malmo, Sweden for biomarker detection using Olink PEA platform (Olink Proteomics AB, Uppsala). For this study, the panel Target 48 Cytokine was used where 1–10 µl of plasma-EDTA and CSF was used for each well. The panel consists of 45 cytokines and chemokines in total. The full list of mediators, protein names and their corresponding detection ranges are provided in Suppl. Table 2. Analyses were performed according to the manufacturer’s protocol. Investigators were not blinded during analysis. For each inflammatory mediator, data were reported both in NPX units (Normalized Protein eXpression, log2 scale) and in standard concentration units (pg/mL) based on standard curves defined during assay validation. The lower and upper limits of quantification (LLOQ and ULOQ) were provided per analyte, defining the validated quantifiable range.

### Electrochemiluminescence

Biomarker levels in both plasma and CSF were measured at our laboratory using electrochemiluminescence (ECL) immunoassays with the Meso Scale Discovery (MSD: Rockville, MD, USA) V-PLEX Human Proinflammatory Panel 1 and the S-PLEX Human IFN-α2a kit. The V-PLEX panel quantified the following 10 cytokines and chemokines: IFN-γ, IL-2, IL-4, IL-6, IL-8, IL-10, IL-13, IL-1β, TNF-α and IL-12p70. The S-PLEX panel was used specifically for IFN-α2a quantification, to investigate potential type I interferon responses linked to endogenous retrovirus activation. A complete list of mediators, protein names and their corresponding detection ranges is provided in Suppl. Table 3. 

All procedures were performed according to the manufacturer’s protocol. Investigators were not blinded during analysis. Before analysis, 60 µl of the SULFO-TAG-labeled antibody mix for each panel was diluted in 2400 µl of the recommended diluent. For both V-PLEX and S-PLEX assays, 50 µl of each sample or standard was loaded into designated wells of 96-well MULTI-SPOT plates and incubated for two hours at room temperature with shaking. Plates were then washed three times using phosphate-buffered saline (PBS) containing 0.05% Tween-20. Subsequently, 25 µl of the prepared detection antibody solution was added and incubated for another two hours. Following a final wash cycle, 150 µl of 2× Read Buffer T was added to each well.

Plates were analyzed using the MESO QuickPlex SQ 120 instrument (MSD), and protein concentrations were calculated from standard curves generated from serial dilutions of known calibrators. All samples were run in duplicates and the mean concentration was used for analysis.

### Cross-Platform Analyses

Nine analytes were measured on both platforms—IFN-γ, IL-2, IL-4, IL-6, IL-8, IL-10, IL-13, IL-1β and TNF-α—enabling direct comparison. IL-12p70 and IFN-α2a were assessed by ECL only and 36 analytes by PEA only.

### Data Handling and Processing

To ensure data quality and reliability, all cytokine and chemokine measurements were assessed for their validity based on their respective LLOQ and ULOQ. For the PEA dataset, protein measurements were accompanied by LLOQ and ULOQ values. Samples with missing concentration were excluded from further analysis. Protein concentrations below the LLOQ, were handled with an imputation strategy by which the concentrations were replaced with LLOQ/2 to retain as much information as possible while minimizing bias [13]. Conversely, values exceeding the ULOQ were capped at ULOQ, ensuring that extreme values did not disproportionately influence the results.

The ECL dataset required a similar approach to handle values below LLOQ and above ULOQ. First, all missing cytokine and chemokine concentration values were removed from the dataset. Then, predefined assay-specific LLOQ and ULOQ values were assigned to each protein to determine whether values were within the quantifiable range. Values below the LLOQ were replaced with LLOQ/2, following common imputation practices in biomarker research [13]. Values above the ULOQ were capped at ULOQ to avoid overestimation of protein concentrations.

The number of observations included/excluded in each analysis and dataset is presented in Suppl. Table 4. 

### Statistical Analysis

All statistical analyses were performed using Stata version 18 (Stata Corp, College Station, TX, USA). Normal distributions were assessed using the Shapiro-Wilk test. As most variables significantly deviated from normality (*p* < 0.05), non-parametric tests were applied. Between-group differences (controls vs. TBI) at each time point were tested using the Mann-Whitney U test in plasma and CSF. Within the TBI group, time point 1 (day 1–3) was compared with time point 2 (day 4–8) using the Wilcoxon signed-rank test for paired samples. Paired comparisons between plasma and CSF within the same patient were also performed using the Wilcoxon signed-rank test. All statistical analyses were two-tailed. To account for multiple testing, p-values from between-group analyses were adjusted using the Benjamini–Hochberg false discovery rate (BH-FDR), performed separately for each compartment (plasma and CSF) and platform (ECL and PEA), with both time points included together within each compartment/platform adjustment. P-values from within-TBI paired analyses (plasma vs. CSF) were likewise FDR-adjusted within platform and compartment. An FDR q-value < 0.05 was considered statistically significant. For the inflammatory mediators quantified by both platforms, agreement, and correlation between ECL and PEA was assessed using Spearman rank correlation and Bland-Altman analysis on log-transformed concentrations, with results reported as the mean ratio (bias) and 95% limits of agreement expressed as fold-differences. Concentrations were log-transformed to handle wide dynamic ranges and proportional differences, enabling fold-scale interpretation in correlation plots and Bland–Altman analyses. For spearman correlation analyses, *p* < 0.05 was considered statistically significant.

### Ethics

The study was approved by the Swedish Ethical Review Authority (Dnr 2017/4069; 2017/1049; Dnr 2022-07096-02). All collected samples were stored in a local biobank (# BD27). Patients were pseudonymized once samples were collected. Since all patients had a reduced level of consciousness and were unable to provide consent, informed consent was obtained from the patient´s next of kin. Written informed consent from the controls was obtained from each patient by written and oral instructions prior to the collection of CSF and blood samples.

## Results

### Patient Demographics

The study included 21 patients with severe TBI and 11 control patients. The median age of TBI patients was 33 (range 18–78) years, and 71% were males. The most common radiological findings were acute subdural hematoma (57%) and traumatic intracerebral hemorrhage/contusion (57%). GCS scores was measured as the last pre-intubation assessment recorded at the primary admitting hospital and were most commonly 7–8 (71%). The presence of pupillary abnormalities as well as the radiological assessment is described in Table 1. Control patients had a median age of 46 (range 21–73) years, and 36% were males. The control patients were most often (91%) operated for a tibial fracture. 

**Table 1:** Patient characteristics. Demographic and clinical characteristics of patients with severe traumatic brain injury (TBI: n = 21). Continuous variables are presented as median (range) or (IQR: interquartile range). Percentages are calculated with n = 21 as the denominator. Radiological findings refer to the index CT and are not mutually exclusive, therefore, percentages may sum to >100%

Characteristic	TBI-patients, *n* = 21
Sex, male, n (%)	15 (71)
Age, years, median (range)	33 (range 18–78)
GCS, n (%) * 7–8* * 6* * 5* * 4* * 3*	15 (71) 2 (10) 0 (0) 1 (5) 3 (14)
GCS, motor score, n (%) * 1* * 2* * 3* * 4* * 5*	3 (14) 1 (5) 0 (0) 2 (10) 15 (71)
Pupillary status on admission, n (%) * Normal* * Unilateral dilated* * Bilateral dilated*	14 (66) 5 (24) 2 (10)
Radiological findings, n (%) * tSAH* * Contusion* * EDH* * ASDH* * Diffuse brain swelling*	8 (38) 12 (57) 5 (24) 12 (57) 1 (5)
Neurosurgical intervention, n (%) * Hematoma evacuation* * Craniectomy* * ICP monitoring*	21 (100) 11 (52) 6 (29) 20 (95)
NICU length of stay, days, median (IQR)	12 (7–19)
Infection during study period, n (%) * Aspiration pneumonia* * Urinary tract infection*	3 (14) 2 (10) 1 (5)
ISS, median (IQR)	23 (18–26)
AIS head, median (IQR)	5 (4–5)
GOS-E 9–12 months post-injury, median (IQR)	4-5 (1–6)
Rotterdam CT Score, n (%) * 3* * 4* * 6*	3 (14) 12 (57) 6 (29)
Trauma mechanism, n (%) * Car accident* * Bike accident* * Fall* * Assault*	4 (19) 3 (14) 10 (48) 4 (19)

*TBI *traumatic brain injury, *GCS *Glasgow Coma Scale, *tSAH* traumatic subarachnoid hemorrhage, *EDH* epidural hematoma, *ASDH* acute subdural hematoma, *ICP *intracranial pressure, *NICU* neuro-intensive care unit, *ISS* Injury Severity Score, *AIS *Abbreviated Injury Scale, *GOS-E* Glasgow Outcome Scale–Extended, *IQR *interquartile range

### Patient Sampling

We aimed to obtain repeated samples from each TBI patient at day 1–3 ( time point 1) and day 4–8 (time point 2) in both plasma and CSF for all TBI patients (*n* = 21). In total, plasma could be obtained for 20/21 at time point 1, 20/21 at time point 2, and 19 patients had plasma samples from both time points. CSF was obtained in 9 patients overall, with CSF samples from both time points in 7 patients. One patient received an EVD at post-injury day 8 and was sampled only at time point 2 for CSF and plasma. All 11 controls provided single-time-point plasma and lumbar CSF.

### Proximity Extension Assay (PEA)

#### Inflammatory Mediator Levels in Plasma Analyzed by PEA Only

**Fig. 1:**
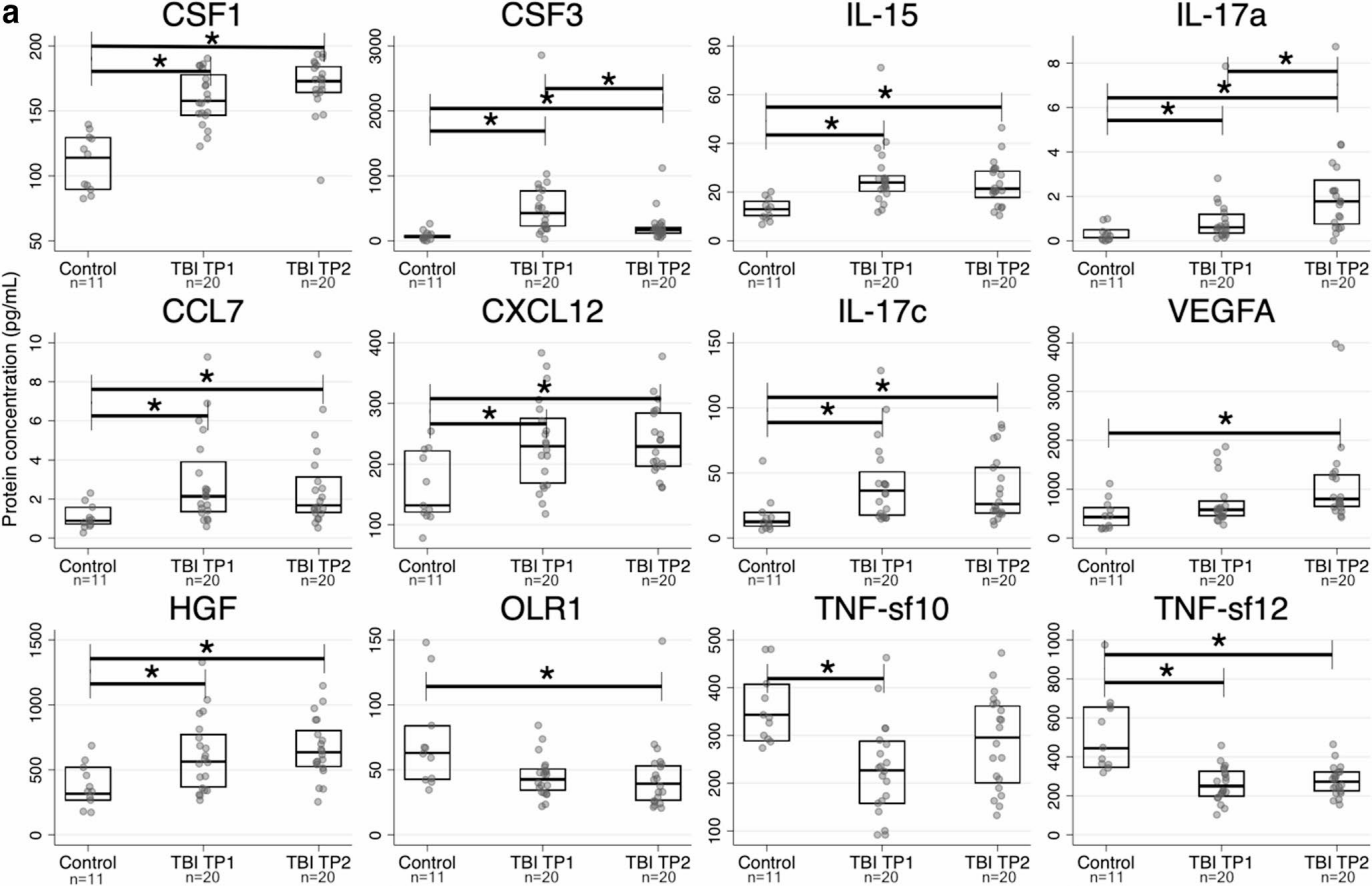

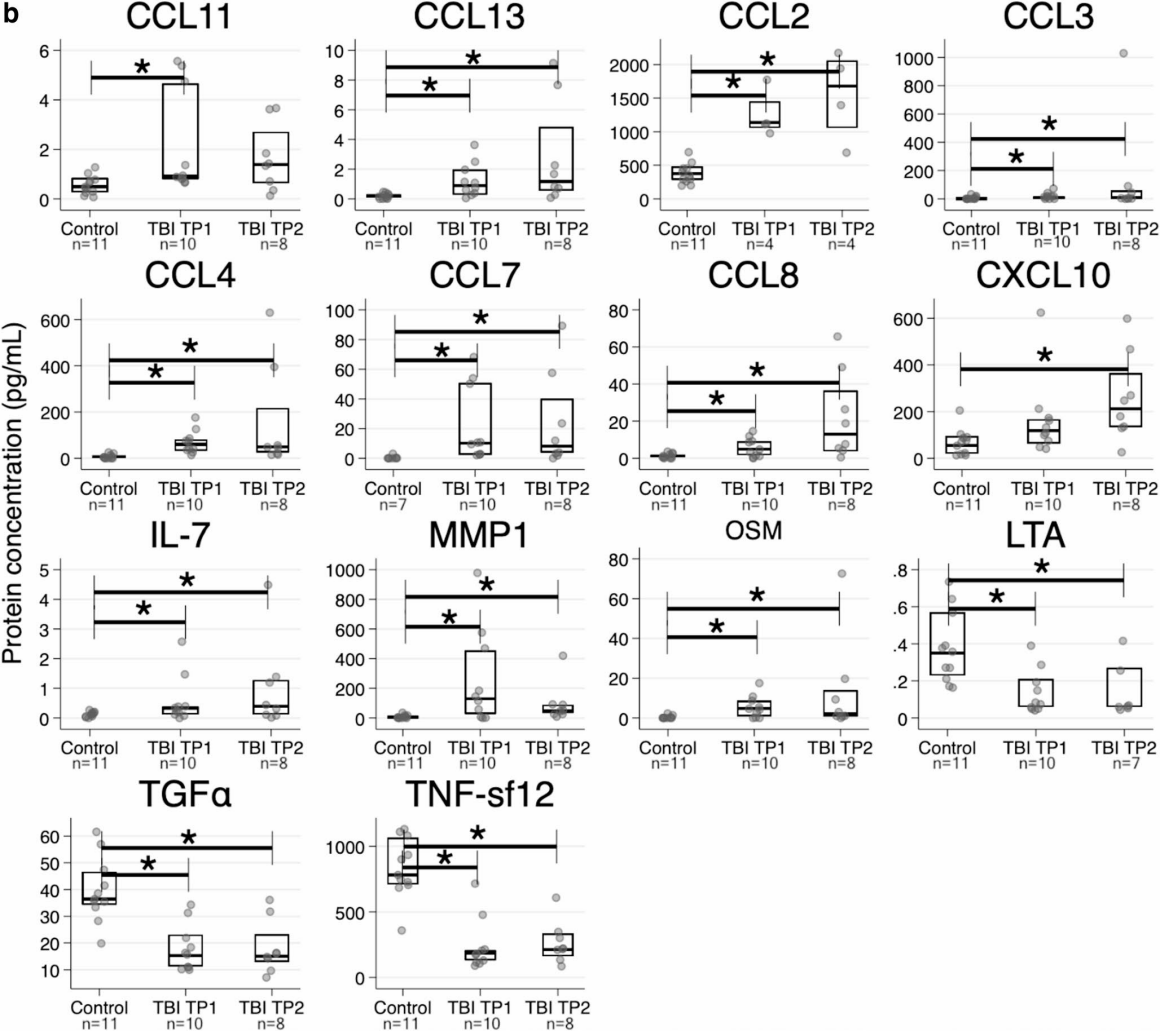
**a**. Levels of inflammatory mediator in plasma - analyzed by PEA platform. Boxplots displaying inflammatory mediator concentrations in plasma samples measured only using the proximity extension assay (PEA) platform. Only cytokines and chemokines that exhibited statistical significance in any comparison are included. The x-axis of each graph represents different groups (Control, TBI TP1: early time point, day 1-3, TBI TP2: later time point, day 4-8), while the y-axis shows inflammatory mediator concentrations in pg/mL. Notably, 9 were increased while 3 were decreased in TBI patients. Boxplots show the median and interquartile range (25th–75th percentiles), with individual observations overlaid and sample size (n) for each compartment is indicated in each panel. Significant differences between groups after BH–FDR correction (q < 0.05) are indicated by *. **b**. Levels of inflammatory mediator in CSF - analyzed by PEA platform. Boxplots displaying inflammatory mediator concentrations in cerebrospinal fluid (CSF) samples measured only using the proximity extension assay (PEA) platform. Only cytokines and chemokines that exhibited statistical significance in any comparison are included. The x-axis of each graph represents different groups (Control, TBI TP1: early time point, day 1-3, TBI TP2: later time point, day 4-8), while the y-axis shows inflammatory mediator concentrations in pg/mL. Notably, 11 were increased while 3 were decreased in TBI patients. Boxplots show the median and interquartile range (25th–75th percentiles), with individual observations overlaid and sample size (n) for each compartment is indicated in each panel. Significant differences between groups after BH–FDR correction (q < 0.05) are indicated by *

In plasma, several inflammatory mediators showed significant differences between TBI patients and controls. Of these, 9 were increased and 3 were decreased at any time point post-injury compared to controls. Mediators that were elevated at both time points included CSF1, CSF3, IL-15, IL-17a, CCL7, CXCL12, IL-17c and HGF. The mediator VEGFA was only elevated at the late time point e.g., day 4–8. Notably, temporal changes were observed in several mediators. CSF3 was significantly higher at the early time point compared to the late time point, whereas IL-17a levels were higher at the late time point compared to early time point. Some mediators showed decreased concentrations in TBI patients compared to controls. OLR1 was reduced in late time point, while TNFsf10 was significantly decreased only at the early time point. TNFsf12 showed consistent reduced levels in both time points. Significant inflammatory mediator concentrations in plasma analyzed by PEA only are presented in Fig. 1a. A summary of significant temporal changes in plasma, including direction of change across time points, is provided in Suppl. Table 5.

The following mediators were unchanged in plasma compared to controls following TBI in the PEA platform: IL-18, CCL19, CCL2, MMP12, LTA, FLT3LG, IL-2, IL-17f, IL-1β, CXCL10, IL-33, TSLP, IFN-γ, CCL4, TGFα, IL-13, CCL8, CCL13, CSF2, IL-4, OSM, MMP1, EGF, IL-7, CXCL9, CXCL11, CCL11, CCL3 and IL-27 (data not shown).

#### Inflammatory Mediator Levels in CSF Analyzed by PEA Only

In CSF, several mediators were significantly different between TBI patients and controls. Of these, 11 were increased, and 3 were decreased at any time point post-injury when compared to controls. Cytokines and chemokines that were consistently elevated across both time points included CCL13, CCL2, CCL3, CCL4, CCL7, CCL8, IL-7, MMP1 and OSM. None of these showed significant temporal changes within the TBI group. The mediator CCL11 was only elevated in early time point and CXCL10 was only elevated in the late time point. Some mediators had significantly lower levels in TBI patients compared to controls. LTA, TGFα and TNFsf12 remained significantly lower across both time points. Significantly altered inflammatory mediator concentrations in CSF analyzed by PEA are presented in Fig. 1b. A summary of significant temporal changes in CSF, including direction of change across time points, is provided in Suppl. Table 6.

The following mediators remained unchanged in CSF compared to controls following TBI in the PEA platform: IL-18, HGF, CCL19, MMP12, FLT3LG, TNFα, IL-17a, IL-2, IL-17f, CSF3, IL-1β, OLR1, VEGFA, IL-33, TSLP, IFN-γ, IL-13, CSF2, IL-4, TNF-sf10, EGF, IL-15, CSF1, CXCL9, CXCL11, IL-17 C, CXCL12 and IL-27 (data not shown).

### Electrochemiluminescence (ECL)

#### Inflammatory Mediator Levels in Plasma and CSF Analyzed by ECL Only

Two analytes, IL-12p70 and IFN-α2a were analyzed by ECL only. IL-12p70 levels were not significantly altered in TBI patients compared to controls in either plasma or CSF at any time point (data not shown). The levels of IFN-α2a in plasma and CSF were all below the LLOQ and, consequently, no further statistical analysis was performed.

### Comparative Analyses (ECL vs. PEA)

#### Inflammatory Mediator Levels in Plasma - Analyzed by Both ECL and PEA

In plasma, significantly higher levels of IL-6, IL-8, IL-10 and TNF-α in TBI patients were seen in ECL analysis at both the early and late time points when compared to controls. IL-6 also showed significantly higher levels at the early time point when compared to the late time point.

**Fig. 2:**
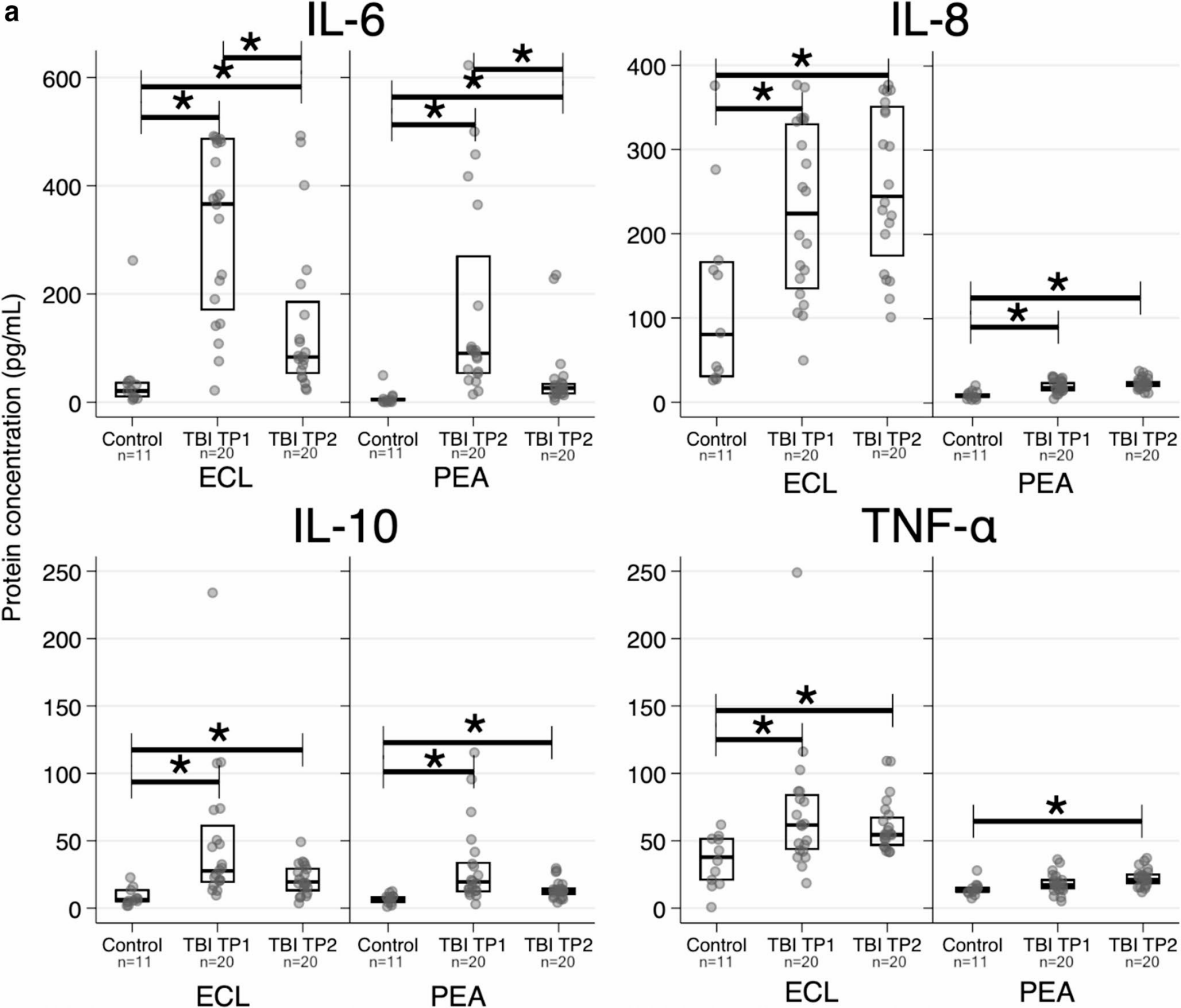

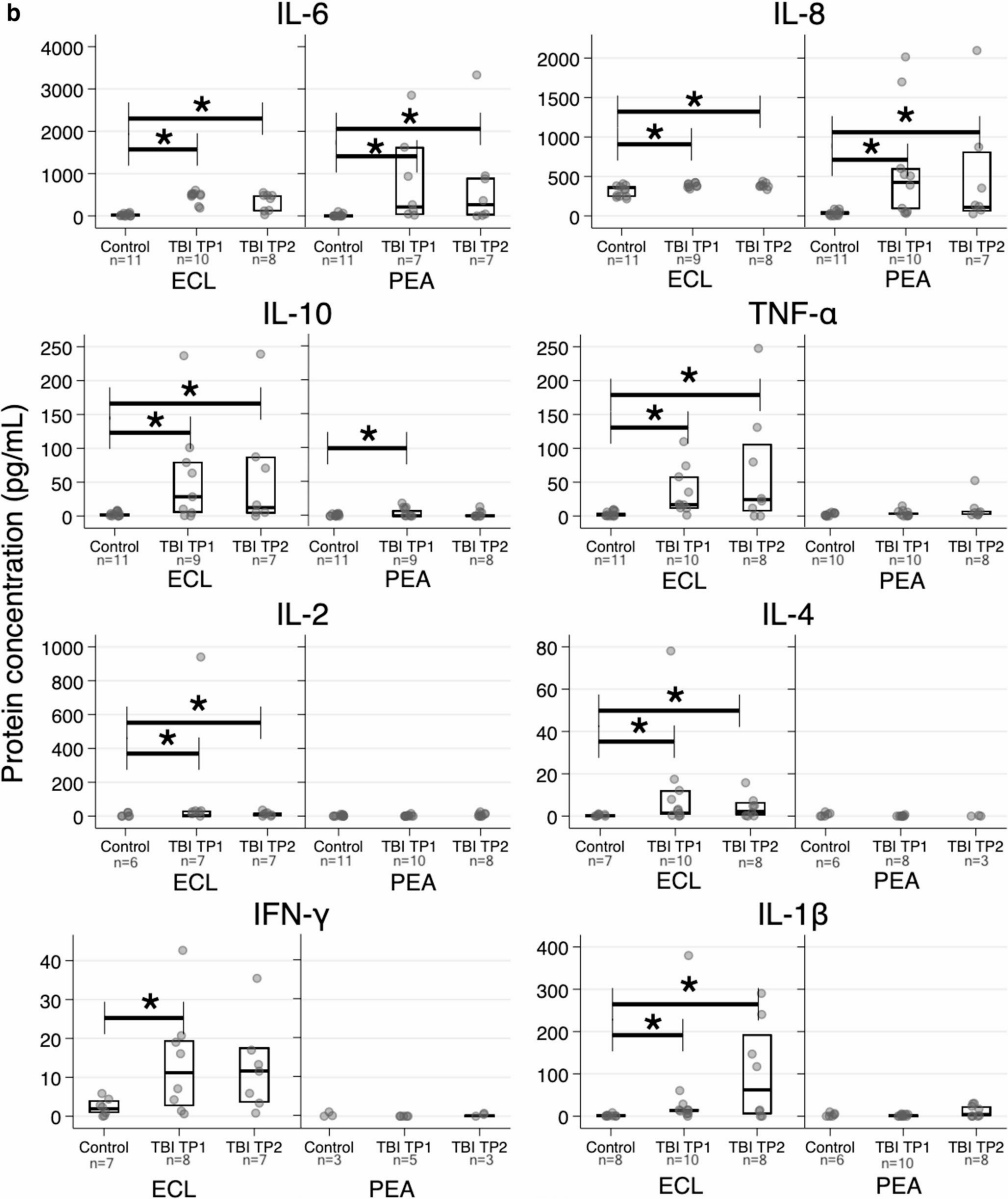
**a**. Levels of Inflammatory mediator in plasma analyzed by both the ECL and PEA platforms. Boxplots displaying inflammatory mediator concentrations in plasma samples measured using both ECL and PEA. Only cytokines and chemokines that exhibited statistical significance in any comparison are included. The x-axis of each graph represents different groups (Control, TBI TP1: early time point, day 1–3, TBI TP2: later time point, day 4–8), and platforms are separate, while the y-axis shows inflammatory mediator concentrations in pg/mL. Boxplots show the median and interquartile range (25th–75th percentiles), with individual observations overlaid and sample size (n) for each compartment is indicated in each panel. Significant differences between groups after BH–FDR correction (q < 0.05) are indicated by *. **b**. Levels of inflammatory mediator in CSF analyzed by both ECL and PEA platform. Boxplots displaying cytokine and chemokine concentrations in cerebrospinal fluid (CSF) samples measured using electrochemiluminescence (ECL) and proximity extension assay (PEA). Only cytokines and chemokines that exhibited statistical significance in any comparison are included. The x-axis of each graph represents different groups (Control, TBI TP1: early time point, day 1–3, TBI TP2: later time point, day 4–8), and platforms are separate, while the y-axis shows inflammatory mediator concentrations in pg/mL. Boxplots show the median and interquartile range (25th–75th percentiles), with individual observations overlaid and sample size (n) for each compartment is indicated in each panel. Significant differences between groups after BH–FDR correction (q < 0.05) are indicated by *

In the PEA platform IL-6, IL-8, and IL-10 were significantly elevated in TBI patients at both time points. IL-6 showed significantly higher values at the early time point compared to the late time point. TNF-α levels were only significantly elevated at the late time point in PEA platform. The levels in plasma of IL-2, IL-4, IL-13, IFN-γ and IL-1β were not significantly altered by TBI on any platform (data not shown). Inflammatory mediator levels in plasma analyzed by both ECL and PEA are presented in Fig. 2a. A summary of significant temporal changes in plasma, including direction of change across time points, is provided in Suppl. Table 5.

#### Inflammatory Mediator Levels in CSF - Analyzed by ECL and PEA

In CSF, ECL analysis showed significantly elevated levels of IL-6, IL-8, IL-10, TNF-α, IL-2, IL-4 and IL-1β in TBI patients at both early and late time points. IFN-γ levels were only significantly elevated at the late time point but not at the early time point.

PEA analysis showed significantly elevated levels at both time points for IL-6, and IL-8, while IL-10 levels were only significantly elevated at the early time point. The levels of IL-13 were not significantly altered by TBI on any platform (data not shown). Inflammatory mediator levels in CSF analyzed by both ECL and PEA are presented in Fig. 2b. A summary of significant temporal changes in CSF, including direction of change across time points, is provided in Suppl. Table 6.

### Correlations Between ECL and PEA Platforms

**Fig. 3:**
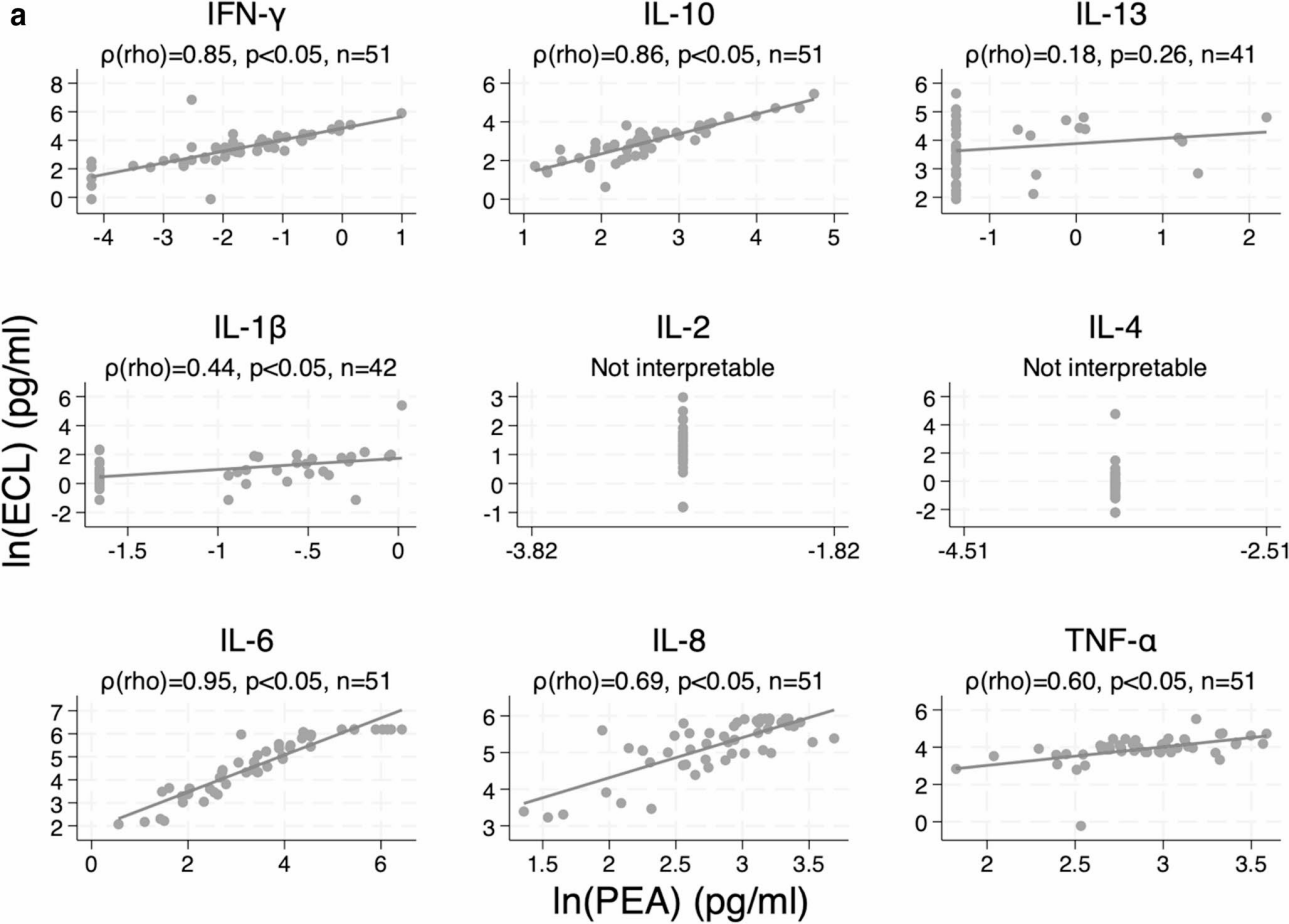

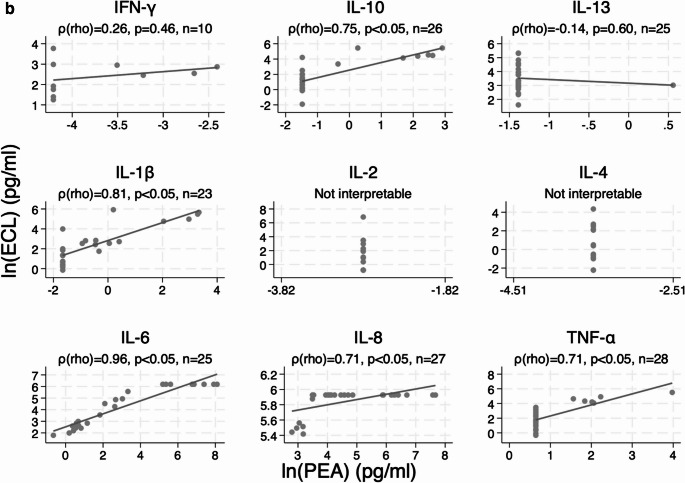
**a**. Correlations for inflammatory mediators in plasma in TBI patients between the ECL and PEA platform. Scatter plots with fitted regression lines illustrating the Spearman correlation between electrochemiluminescence (ECL) and proximity extension assay (PEA) measurements for nine overlapping mediators in plasma samples. Each dot represents an individual sample. Spearman’s correlation coefficient (ρ, rho), corresponding p-value and sample size (n) are shown in each panel. Concentrations are displayed on a logarithmic scale on both axes (log-transformed ECL on the y-axis and log-transformed PEA on the x-axis). Significant correlations were observed for IFN-γ, IL-10, IL-1β, IL-6, IL-8, and TNF-α, with the strongest correlations for IL-6 (ρ = 0.95), IL-10 (ρ = 0.86), and IFN-γ (ρ = 0.85). IL-2 and IL-4 displayed artificial correlations due to no variability in PEA measurements and were therefore labeled “not interpretable”. **b**. Correlations for inflammatory mediators in CSF in TBI patients between the ECL and PEA platforms. Scatter plots with fitted regression lines illustrating the Spearman correlation between electrochemiluminescence (ECL) and proximity extension assay (PEA) measurements for nine overlapping mediators in CSF samples. Each dot represents an individual sample. Spearman’s correlation coefficient (ρ, rho), corresponding p-value and sample size (n) are shown in each panel. Concentrations are displayed on a logarithmic scale on both axes (log-transformed ECL on the y-axis and log-transformed PEA on the x-axis). Significant correlations were observed for IL-10, IL-1β, IL-6, IL-8, and TNF-α. Strong correlations were seen for IL-6 (ρ = 0.96), IL-1β (ρ = 0.81), IL-10 (ρ = 0.75), IL-8 (ρ = 0.71) and TNF-α (ρ = 0.71). IL-2 and IL-4 displayed artificial correlations (due to no variability in PEA measurements and were therefore labeled “not interpretable”)

**Fig. 4:**
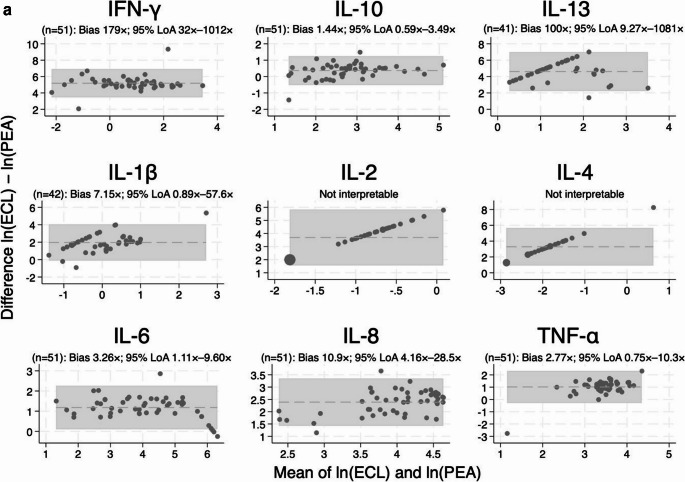

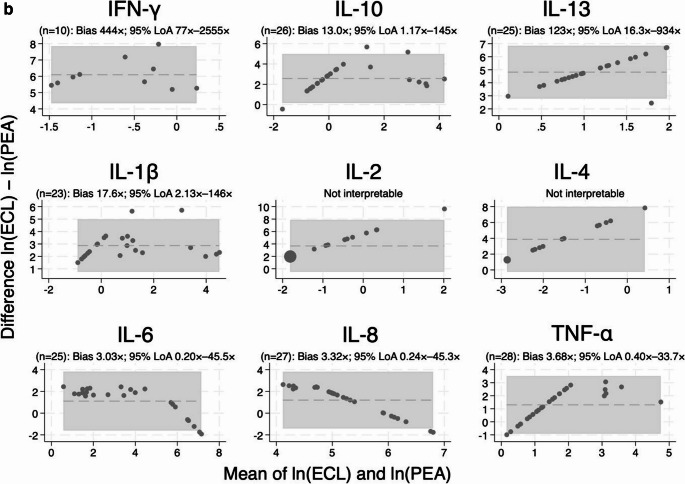
**a**. Bland–Altman agreement between ECL and PEA in plasma for TBI patients (log scale). Bland–Altman difference plots for nine overlapping mediators in plasma samples using electrochemiluminescence (ECL) and proximity extension assay (PEA). Concentrations were log-transformed prior to analysis. The x-axis shows the mean of ln(ECL) and ln(PEA), and the y-axis shows the difference ln(ECL) − ln(PEA). The central horizontal line indicates the mean log-difference (bias). The upper and lower 95% limits of agreement (bias ± 1.96 × SD of the differences) define the shaded region, which represents the interval within which 95% of paired differences are expected to fall. For interpretability on the original scale, the bias and limits are also presented as fold-differences (ratios). Each point corresponds to one paired plasma sample (n shown in each panel), wider limits indicate poorer agreement and/or greater proportional error across the measurement range. IL-2 and IL-4 are labeled “not interpretable” because PEA values showed no variability/tied values, making the Bland-Altan bias estimates unreliable. **b**. Bland–Altman agreement between ECL and PEA in CSF for TBI patients (log scale). Bland–Altman difference plots for nine overlapping mediators in CSF samples using electrochemiluminescence (ECL) and proximity extension assay (PEA). Concentrations were log-transformed prior to analysis. The x-axis shows the mean of ln(ECL) and ln(PEA), and the y-axis shows the difference ln(ECL) − ln(PEA). The central horizontal line indicates the mean log-difference (bias). The upper and lower 95% limits of agreement (bias ± 1.96 × SD of the differences) define the shaded region, which represents the interval within which 95% of paired differences are expected to fall. For interpretability on the original scale, the bias and limits are also presented as fold-differences (ratios). Each point corresponds to one paired CSF sample (n shown in each panel), wider limits indicate poorer agreement and/or greater proportional error across the measurement range. IL-2 and IL-4 are labeled “not interpretable” because PEA values showed no variability/tied values, making the Bland-Altman bias estimates unreliable

When comparing overlapping inflammatory mediators between ECL and PEA, nine cytokines and chemokines were analyzed using both techniques (IFN-γ, IL-10, IL-13, IL-1β, IL-2, IL-4, IL-6, IL-8, and TNF-α). Spearman correlation analysis was conducted separately for plasma and CSF samples for both TBI and control groups. IL-2 and IL-4 were included for completeness but labeled not interpretable due to tied values (minimal variability), which yield artificial correlations. Scatter plots (log-transformed concentrations) are shown in Fig. 3a (plasma) and Fig. 3b (CSF). Agreement between platforms was further assessed using Bland–Altman analysis on log-transformed concentrations, with results reported as fold-differences (ratio bias) and 95% limits of agreement (LoA) (Fig. 4a–b).

#### Correlations Between the ECL and PEA Platform in Plasma Samples

In plasma, strong and statistically significant correlations between the two platforms were observed for IL-6 (ρ = 0.95), IL-10 (ρ = 0.86), IFN-γ (ρ = 0.85), IL-8 (ρ = 0.69), TNF-α (ρ = 0.60). IL-1β (ρ = 0.44) showed moderate yet significant correlations. IL-13 showed weak non-significant correlation (ρ = 0.18, *p* = 0.26), while IL-2 and IL-4, they were excluded due to lack of variability and artificially inflated correlation values, caused by tied ranks (Fig. 3a). Bland–Altman analysis in plasma demonstrated proportional differences between platforms for several analytes. Ratio bias was smallest for IL-10 (bias 1.44×; 95% LoA 0.59×–3.49×), TNF-α (2.77×; 0.75×–10.3×), IL-6 (3.26×; 1.11×–9.60×), IL-1β (7.15×; 95%; 0.89×–57.6×) and IL-8 (10.9×; 4.16×–28.5×), whereas IFN-γ (179×; 32×–1012×) and IL-13 (100×; 9.27×–1081×) showed markedly wider limits of agreement (Fig. 4a).

#### Correlations and Agreement Between the ECL and PEA Platform in CSF Samples

In CSF, the strongest correlation was observed for IL-6 (ρ = 0.96, *p* = 0.05) followed by IL-1β (ρ = 0.81), IL-10 (ρ = 0.75), TNF-α (ρ = 0.71), and IL-8 (ρ = 0.71), all statistically significant. IFN-γ (ρ = 0.26, *p* = 0.46) and IL-13 (ρ = − 0.14, *p* = 0.60) showed weak and non-significant correlation due to low variability and IL-2 and IL-4 were again excluded due to tied values (Fig. 3b). Bland–Altman analysis in CSF showed variability in agreement for several analytes compared. Ratio bias was smallest for IL-6 (bias 3.03×; 95% LoA 0.20×–45.5×), IL-8 (3.32×; 0.24×–45.3×), and TNF-α (3.68×; 0.40×–33.7×), while IL-10 (13.0×; 1.17×–145×), IL-1β (17.6×; 2.13×–146×), IL-13 (123×; 16.3×–934×), and IFN-γ (444×; 77×–2555×) demonstrated large proportional bias and wide 95% LoA, indicating limited absolute agreement despite moderate-to-strong correlations for some analytes (Fig. 4b).

#### Platform Variability

Across the nine overlapping analytes, platform-dependent differences emerged, both by compartment and time.

Compartment differences: In the ECL platform TNF-α, IL-2, IL-4, IFN-γ and IL-1β were significantly increased in CSF however this was not seen in the PEA platform. TNF-α was only increased in plasma in the PEA platform however in ECL it was increased in both plasma and CSF.

Temporal differences: In plasma, TNF-α was increased at both time points in ECL however only at the late time point in PEA. In CSF, IL-10 was increased at both time points in ECL, but only at early time point by PEA.

### Correlation Between Plasma and CSF

#### Unique Changes in Plasma

**Fig. 5:**
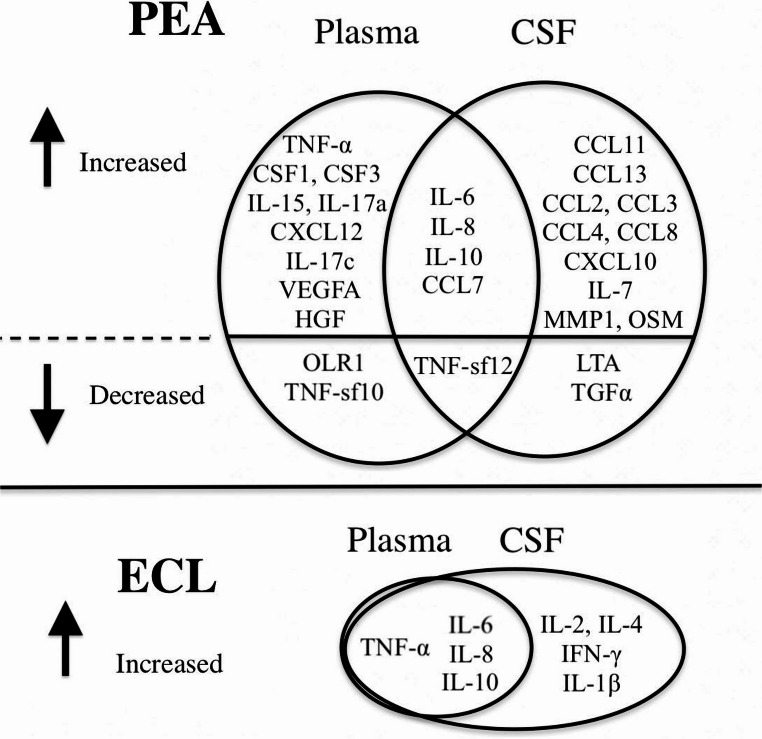
Venn diagrams showing mediators significantly altered in plasma and CSF following TBI assessed separately for the PEA and ECL platforms. Significant mediators are grouped by direction of change—either increased or decreased—within each biological compartment, plasma or cerebrospinal fluid (CSF). Overlapping areas represent mediators altered in both plasma and CSF. The top panel presents results from the proximity extension assay (PEA) platform, while the bottom panel shows electrochemiluminescence (ECL) platform. In the PEA panel, IL-6, IL-8, IL-10 and CCL7 were significantly elevated in both plasma and CSF, while TNFsf12 was decreased in both compartments. In the ECL panel, TNF-α, IL-6, IL-8 and IL-10 were significantly increased in both plasma and CSF.

Several mediators showed compartment-specific alterations in plasma but were not significantly changed in CSF. In the PEA analysis, significant increases were observed for: TNF-α, CSF1, CSF3, IL-15, IL-17a, CXCL12, IL-17c, VEGFA and HGF compared to controls and compared with CSF. Additionally, OLR1 and TNFsf10 levels were significantly decreased in plasma. In the ECL analysis, no mediator was uniquely changed in plasma compared to controls and CSF. (Fig. 5)

#### Unique Changes in CSF

In the CSF compartment, a distinct set of mediators was significantly altered, but not changed in plasma. In the PEA analysis, the level of the following mediators was significantly increased: CCL11, CCL13, CCL2, CCL3, CCL4, CCL8, CXCL10, IL-7, MMP1 and OSM compared to controls and plasma. Additionally, LTA and TGFα were significantly decreased compared to plasma. In the ECL analysis, IL-2, IL-4, IFN-γ and IL-1β were uniquely increased in CSF but not in plasma. (Fig. 5)

### Altered and Overlapping Changes in Plasma and CSF

Several mediators were significantly altered in both plasma and CSF, suggesting a shared or systemic component of the inflammatory response following TBI. In the PEA analysis, overlapping increased mediators included IL-6, IL-8, IL-10 and CCL7. TNFsf12 was decreased in both compartments. In the ECL panel, TNF-α, IL-6, IL-8 and IL-10 were the only mediators significantly increased in both compartments. A Venn diagram summarizing the overlapping and unique mediator changes in plasma and CSF is presented in Fig. 5 and a table compiling these changes can be seen in Suppl. Table 7.

#### Comparison of Mediator Levels Between CSF and Plasma – PEA

Among the mediators that were significantly elevated in both plasma and CSF, several of them demonstrated clear differences in compartmental expression. IL-6, IL-8 and CCL7 showed significantly higher concentrations in CSF compared to plasma. In contrast, IL-10 was significantly higher in plasma. TNFsf12 showed no significant compartmental differences. These findings can be visualized in Suppl. Figure 1.

#### Comparison of Mediator Levels Between CSF and Plasma - ECL

In the ECL analysis, IL-6 levels and IL-8 were significantly higher in CSF compared to plasma (*p* < 0.05). TNF-α and IL-10 showed no significant compartmental differences. These findings can be visualized in Suppl. Figure 2.

## Discussion

Inflammatory activation—particularly involving IL-6, IL-8 and IL-10—has been widely reported in both plasma and CSF after severe TBI [4]. In the present study, we extend this work by profiling inflammatory mediators in parallel in both plasma and CSF across the first post-injury week using two analytical platforms (ECL and PEA). We observed consistent elevations of IL-6, IL-8 and IL-10 in both compartments, alongside additional cytokines and chemokines showing compartment- and time-dependent patterns, supporting partially overlapping but compartmentalized systemic and central inflammatory responses after TBI. The observed inflammatory activation is likely a consequence of direct tissue injury, blood-brain barrier (BBB) disruption, and mechanisms that trigger innate immune signaling in both the CNS and the periphery [14]. The orchestration of this inflammatory response is complex and involves a wide range of mediators, including pro- and anti-inflammatory cytokines and chemokines. Notably, inflammation can exert both beneficial and detrimental effects in TBI, but the underlying mechanisms driving this dual role remain poorly understood [15]. Evaluating the two different platforms, ECL and PEA, we showed a variable correlation and measured concentrations between the analytical methods, stressing the importance of awareness when using different techniques to assess post-injury inflammation.

Besides showing several mediators that were affected in both compartments, our study also reveals a unique pattern of mediators only affected in plasma and CSF, respectively. The PEA analysis identified unique increase in the following plasma mediators: TNF-α, CSF1, CSF3, IL-15, IL-17a, CXCL12, IL-17c, VEGFA and HGF. On the contrary OLR1 and TNF-sf10 were decreased only in plasma. These changes may contribute to the regulation of specific regulatory or homeostatic pathways and support previous findings showing temporal shifts in systemic mediators post-injury in both human and animal models [16, 17]. This plasma inflammatory signature could partly reflect systemic contributors such as extracranial injury or intercurrent infection. Although overt infection was uncommon in the evaluated patients during the sampling period, the TBI cohort had substantial overall trauma burden mainly due to their head injury (median ISS 23, median AIS head 5). The systemic injuries may have contributed to circulating mediator levels, as suggested in prior work linking higher IL-6, IL-8, IL-15 to worse outcome [6]. In CSF both platforms revealed increased IL-6, IL-8 and IL-10 in TBI patients, with some differences in the temporal profile. In addition, the PEA platform identified a group of mediators that were uniquely elevated in CSF, including: CCL11, CCL13, CCL2, CCL3, CCL4, CCL8, CXC10, IL-7, MMP1 and OSM. Furthermore, LTA and TGFα were uniquely decreased in CSF. In the ECL platform IL-1β, IL-2, IL-4, and IFN-γ was uniquely elevated in CSF, although not captured by the PEA platform. Overall, these findings support compartmentalized neuroinflammation after TBI, in line with prior CSF and microdialysis studies [18, 19]. These findings highlight that while some mediators reflect a common inflammatory response across compartments, others appear to be predominantly generated within the CNS. Inflammatory mediators that are present in both plasma and CSF could also have different source and functional impact [20]. While blood sampling is more easily accessible, CSF likely represents a more direct window into neuroinflammatory processes. After TBI, BBB permeability increases and blood-borne molecules can enter the parenchyma, while signals released by the TBI itself (e.g., cytokines such as TNF-α) further amplify BBB permeability, meaning CSF cytokine levels may reflect a mixture of central production and plasma ingress [21, 22]. 

We observed that some inflammatory mediators were decreased in TBI patients compared to controls. In the PEA analysis, OLR1 and TNF-sf10 were decreased in plasma, while LTA and TGFα were decreased in CSF. TNFsf12 was consistently reduced in both plasma and CSF. These divergences may reflect early post-TBI immunosuppression occurring alongside compartmentalized central inflammation [23]. However, alternative explanations such as assay differences or sampling-window effects cannot be excluded.

In our study, we compared CSF from an external ventricular drain to CSF obtained via lumbar drainage in controls. There are obvious ethical concerns against obtaining ventricular CSF from controls, yet it is important to acknowledge that protein composition may vary between these compartments (e.g., due to CSF gradients and compartmental dynamics), which may influence biomarker interpretation [24, 25]. 

Although our control group consisted of orthopedic patients with minor extremity injuries, we cannot exclude that certain inflammatory mediators may have been elevated in controls compared to healthy and uninjured individuals, as suggested in previous work [5, 26]. 

Multiplex platforms enable broad, simultaneous profiling of inflammatory proteins after TBI, but the resulting high-dimensional data require careful, data-driven selection of the most informative mediators and patterns [27]. Conversely, from large datasets much work is needed to select what factors, and patterns, contribute most to the secondary insult [28, 29]. While cytokines and chemokines are often labeled as pro- or anti-inflammatory, such categorization is increasingly recognized as an oversimplification. Following TBI, mediators such as IL-6 and TNF-α rise early and are implicated in BBB disruption, excitotoxicity, and immune-cell recruitment, and are often associated with worse outcome [30–32]. However, the inflammatory response may also have protective or regenerative roles in certain contexts. For instance, even though IL-6 has been linked to increased ICP and increased neuronal damage, elevated IL-6 levels have also been associated with better one-year neurological outcome [33, 34]. Similarly, TNF-α may worsen injury acutely but support neuroprotection during later phases, as shown in knockout mouse models [35]. IL-10 is classically viewed as anti-inflammatory, but may also display complex mechanisms of action. It inhibits pro-inflammatory cytokine production and limits immune cell activation [36]. IL-10 levels have been associated with both improved outcomes in animal models and worse prognosis in clinical TBI studies [31, 32, 37]. 

Separately, we evaluated the IFN-α2a response using the ECL platform since IFN-α can be activated secondary to endogenous and selective retroviral activation and be elevated following severe TBI [8]. However no changes were detected post-injury in TBI patients. It remains uncertain whether IFN-α2a is suitable as a biomarker for post-TBI retroviral-induced inflammation, whether any IFN-α response occurs in a narrow temporal window outside our sampling period, or whether levels remain below detection thresholds.

A key aim of this study was to compare ECL and PEA for overlapping inflammatory mediators. Spearman correlations showed good rank-order agreement for several analytes, particularly IL-6, IL-10, IFN-γ, IL-8 in plasma, and IL-6, IL-1β, IL-10 in CSF. IL-13 showed poor inter-platform agreement, largely attributable to low abundance and minimal variability between platforms, and IL-2 and IL-4 showed no variability and could not be further interpreted [38]. In addition, the relatively small sample size limits statistical power, which contribute to some correlations not reaching significance. Our findings align with prior multi-platform comparisons, where most overlapping proteins showed strong correlations (ρ > 0.8), while low-abundance analytes with frequent values below the detection limit (e.g., IL-2, IL-4, IL-5, IL-13) demonstrated poor agreement [5]. However, our Bland–Altman analyses demonstrated that correlation did not equate to interchangeability, with several mediators showing substantial proportional bias and wide 95% limits of agreement, indicating meaningful differences in absolute concentrations between platforms. We also observed platform-dependent differences by compartment. For example, TNF-α was elevated in both plasma and CSF in ECL but only in plasma in PEA. Moreover, IL-2, IL-4, IFN-γ and IL-1β were increased in CSF on ECL but showed no increase on PEA. Arguably, this should not be interpreted as “higher accuracy” *per se*, rather it could reflect platform- and matrix-dependent detectability (e.g., performance near LLOQ), differences in antibody pairs/epitopes, and calibration approaches. From a practical biomarker perspective, ECL is often advantageous when the aim is robust quantification of a smaller, well-defined cytokine set, with good sensitivity and straightforward in-house implementation (as in our lab), and with results anchored to standard-curve concentrations. In contrast, PEA is advantageous when the goal is broad, multiplexed discovery-style profiling across many proteins with low sample volume and standardized workflows, particularly when run in an experienced core facility. In our setting, PEA required outsourcing to a specialized laboratory, offered substantially wider biomarker coverage, and was more resource-intensive, whereas ECL was more accessible locally and allowed closer hands-on oversight. When results diverge, we recommend interpreting findings more cautiously, especially when one platform is near LLOQ and give greater weight to signals that are clearly quantifiable (well above LLOQ) and concordant in direction across platforms - as such concordance increases confidence that the change reflects a true biological effect.

This underscores that conclusions about which mediators are significantly altered may vary depending on platform-specific sensitivity, calibration, and matrix effects [39]. Therefore, cross-platform comparisons should be interpreted cautiously, and greatest confidence placed in signals that replicate across methods [12, 38]. 

## Strengths and Limitations of the Study

Our study provides a dual-compartment and dual-platform evaluation of early post-TBI inflammation, using both CSF and plasma alongside two complementary biomarker technologies, ECL and PEA. In addition, two time points were evaluated. The inclusion of CSF sampling in a clinical TBI cohort has been performed less often than in plasma and allowed for an assessment of central neuroinflammation that was found to be robust. In addition, we directly compared ECL and PEA technologies in the context of severe TBI, providing novel insights into their performance and application in neuroinflammatory profiling.

This exploratory study has several limitations. The sample size was small, which limits statistical power and generalizability. Our TBI cohort was heterogeneous in terms of injury mechanism and severity, with differences in GCS scores at admission that may influence mediator expression. Moreover, while we used two validated platforms, not all mediators were measured across both platforms. Additionally, platform-specific variations in sensitivity and quantification may influence interpretation. Furthermore, although samples were collected systematically within defined timeframes, individual variation in sampling relative to injury may have influenced cytokine levels, especially in the early time point. Sampling time was also variable, and ideally, daily or highly defined sampling intervals are preferred [29, 40]. We did not correlate inflammatory mediators to outcome associations due to limited power. We also did not assess other biological fluids (e.g., microdialysate), which may provide higher spatial although highly focal resolution of central inflammation [18, 34]. The CSF-serum albumin quotient as a proxy for BBB integrity could also have been included [27]. To limit Type I error from multiple testing, between-group p-values were adjusted using Benjamini–Hochberg FDR within each compartment and platform (tests combined across time points). This reduces false positives but may lower power for smaller effects (Type II error), especially with the modest sample size. Finally, it is beyond the scope of this descriptive pilot study to provide a mechanistic in-depth review of each cytokine/chemokine and its pathophysiological implications. Rather, we aimed to generate exploratory insights to guide future hypothesis-driven analyses.

## Conclusions

Our data shows that post-TBI inflammation is both time- and mediator-specific and several analytes displayed compartment-specific behavior, underscoring that central and systemic inflammation only partially overlap. The comparison between ECL and PEA platforms underscores the need for methodological awareness when assessing post-injury cytokine and chemokine levels. Understanding the complex inflammatory dynamics post-TBI is critical for developing targeted, time-sensitive therapeutic strategies. Future research should continue to explore the intricate dynamics of the inflammatory response in TBI.

## Supplementary Information

Below is the link to the electronic supplementary material.

Supplementary Material 1 Proof (DOCX 293 KB)

## Data Availability

Anonymized summary data supporting the figures and tables are included in the article. The raw cytokine/chemokine concentration matrices (PEA and ECL) and analysis scripts are available from the corresponding author upon reasonable request.

## Abbreviations

BBB: blood–brain barrier
CNS: central nervous system
CSF: cerebrospinal fluid
ECL: electrochemiluminescence
EVD: external ventricular drain
GCS: Glasgow Coma Scale
GOS-E: Glasgow Outcome Scale–Extended
ICP: intracranial pressure
IQR: interquartile range
ISS: Injury Severity Score
LLOQ: lower limit of quantification
MSD: Meso Scale Discovery
NICU: Neurointensive Care Unit
NPX: Normalized Protein eXpression
PBS: phosphate-buffered saline
PEA: proximity extension assay
TBI: traumatic brain injury
ULOQ: upper limit of quantification
