# Supplementary material for: A Dynamic and Complex Early Inflammatory Response in Blood and Cerebrospinal Fluid of Severe Traumatic Brain Injury Patients: A Dual Platform Analysis
Source: Inflammation. 2026 Jun 26;49(1):156. doi: 10.1007/s10753-026-02549-9 (PMC13309478; doi:10.1007/s10753-026-02549-9)
Supplement: Supplementary file 1 — Supplementary Material 1 Proof (DOCX 293 KB) [file 10753_2026_2549_MOESM1_ESM.docx]

**Supplemental information**

**Tables**

| **Exclusion criterion** |
| --- |
| Age <18 or >80 years |
| Admission GCS >8 |
| Expected NICU stay <7 days |
| Pre-existing neurodegenerative disease (e.g., Alzheimer’s, Parkinson’s, MS) |
| Pregnancy |
| Patients deemed moribund or receiving palliative/comfort care at admission |

**Suppl. Table 1. Exclusion criterion**. The following exclusion criteria were applied in recruitment of TBI patients to the study, to avoid confounding from pre-existing conditions or limited life expectancy. Abbreviations: GCS: Glasgow Coma Scale. NICU: Neurointensive Care Unit. MS: Multiple Sclerosis. TBI: Traumatic Brain Injury.

| **Gene** | **Protein name** | **LLOQ (pg/mL)** | **ULOQ (pg/mL)** |
| --- | --- | --- | --- |
| IL18 | Interleukin-18 | 0.95 | 15625 |
| HGF | Hepatocyte growth factor | 0.24 | 15625 |
| CCL19 | C-C motif chemokine 19 | 0.12 | 1953 |
| CCL2 | C-C motif chemokine 2 | 0.24 | 3906 |
| MMP12 | Macrophage metalloelastase | 7.63 | 15625 |
| LTA | Lymphotoxin-alpha | 0.12 | 3906 |
| FLT3LG | Fms-related tyrosine kinase 3 ligand | 0.95 | 3906 |
| TNF-α | Tumor necrosis factor | 3.81 | 15625 |
| IL17A | Interleukin-17A | 0.12 | 15625 |
| IL2 | Interleukin-2 |  |  |
| IL17F | Interleukin-17F | 0.24 | 3906 |
| CSF3 | Granulocyte colony-stimulating factor | 7.63 | 62500 |
| IL1β | Interleukin-1 beta | 0.19 | 3125 |
| OLR1 | Oxidized low-density lipoprotein receptor 1 | 0.95 | 3906 |
| TNFsf12 | Tumor necrosis factor ligand superfamily member 12 | 3.81 | 15625 |
| CXCL10 | C-X-C motif chemokine 10 | 0.12 | 1953 |
| VEGFA | Vascular endothelial growth factor A | 0.48 | 7812 |
| IL33 | Interleukin-33 | 0.24 | 7812 |
| TSLP | Thymic stromal lymphopoietin | 0.48 | 7812 |
| IFNG | Interferon gamma | 0.03 | 3906 |
| CCL4 | C-C motif chemokine 4 | 0.12 | 3906 |
| TGFα | Protransforming growth factor alpha | 0.48 | 1953 |
| IL13 | Interleukin-13 | 0.48 | 15625 |
| CXCL8 | Interleukin-8 | 0.24 | 1953 |
| CCL8 | C-C motif chemokine 8 | 0.03 | 1953 |
| IL6 | Interleukin-6 | 0.06 | 3906 |
| CCL13 | C-C motif chemokine 13 | 0.06 | 1953 |
| CSF2 | Granulocyte-macrophage colony-stimulating factor | 0.24 | 7812 |
| CCL7 | C-C motif chemokine 7 | 0.12 | 1953 |
| IL4 | Interleukin-4 | 0.06 | 3906 |
| TNFsf10 | Tumor necrosis factor ligand superfamily member 10 | 0.95 | 7812 |
| OSM | Oncostatin-M | 0.12 | 1953 |
| MMP1 | Interstitial collagenase | 0.95 | 7812 |
| EGF | Pro-epidermal growth factor | 0.48 | 977 |
| IL7 | Interleukin-7 | 0.24 | 1953 |
| IL15 | Interleukin-15 | 0.12 | 15625 |
| CSF1 | Macrophage colony-stimulating factor 1 | 0.12 | 3906 |
| CXCL9 | C-X-C motif chemokine 9 | 0.12 | 3906 |
| CXCL11 | C-X-C motif chemokine 11 | 0.12 | 1953 |
| IL17C | Interleukin-17C | 1.91 | 15625 |
| CXCL12 | Stromal cell-derived factor 1 | 30.52 | 31250 |
| CCL11 | Eotaxin | 0.24 | 15625 |
| IL10 | Interleukin-10 | 0.12 | 31250 |
| CCL3 | C-C motif chemokine 3 | 0.03 | 1953 |
| EBI3_IL27 | Interleukin-27 | 0.24 | 31250 |

**Suppl. Table 2. List of PEA inflammatory mediators.** Inflammatory mediators included in the Olink Target 48 Cytokine panel, analyzed using the Proximity Extension Assay (PEA) platform. The panel consists of 45 cytokines and chemokines in total. Gene symbols, protein names, and the validated lower and upper limits of quantification (LLOQ and ULOQ) in pg/mL are provided according to the manufacturer's specifications.

| **Gene/abbreviation** | **Protein name** | **LLOQ (pg/mL)** | **ULOQ (pg/mL)** |
| --- | --- | --- | --- |
| IFN-γ | Interferon gamma | 1.76 | 938 |
| IL-1β | Interleukin-1 beta | 0.646 | 375 |
| IL-2 | Interleukin-2 | 0.89 | 938 |
| IL-4 | Interleukin-4 | 0.218 | 158 |
| IL-6 | Interleukin-6 | 0.633 | 488 |
| IL-8 | Interleukin-8 (CXCL8) | 0.591 | 375 |
| IL-10 | Interleukin-10 | 0.298 | 233 |
| IL-12p70 | Interleukin-12 p70 subunit | 1.22 | 315 |
| IL-13 | Interleukin-13 | 4.21 | 353 |
| TNF-α | Tumor necrosis factor alpha | 0.69 | 248 |
| IFN-α2a | Interferon alpha 2a | 4.9 fg/mL | 67 000 fg/mL |

**Suppl. Table 3. List of inflammatory mediators analyzed by ECL.**

Inflammatory mediators analyzed using the Meso Scale Discovery (MSD) electrochemiluminescence (ECL) platform. Analytes were measured with the V-PLEX Proinflammatory Panel 1 and the S-PLEX IFN-α2a assay. Gene abbreviations, protein names, and validated lower and upper limits of quantification (LLOQ and ULOQ) in pg/mL are listed according to the manufacturer's specifications.

| **Analysis (dataset)** | **Compartment** | **Units** | **Initial observations/pairs, n** | **Excluded, n (%)** | **Final included, n (%)** |
| --- | --- | --- | --- | --- | --- |
| **Total dataset** | All | Observations | 4480 | 323 (7.2%) | 4157 (92.8%) |
| **PEA dataset** | All  Plasma  CSF | Observations Observations  Observations | 3600  2295  1305 | 159 (4.4%)  16 (0.7%)  159 (12.2%) | 3441 (95.6%)  2279 (99.3%)  1162 (87.8%) |
| **ECL dataset** | All  Plasma  CSF | Observations Observations  Observations | 880  561  319 | 164 (18.6%)  107 (19.1%)  57 (17.9%) | 716 (81.4%)  454 (80.9%)  262 (82.1%) |
| **PEA dataset**   - Controls - TBI TP1 - TBI TP2 | Plasma  Plasma  Plasma | Observations Observations  Observations | 495  945  855 | 3 (0.6%)  6 (0.6%)  7 (0.8%) | 492 (99.4%)  939 (99.4%)  848 (99.2%) |
| **PEA dataset**   - Controls - TBI TP1 - TBI TP2 | CSF  CSF  CSF | Observations  Observations Observations | 495  495  315 | 74 (14.9%)  39 (7.9%)  30 (9.5%) | 421 (85.1%)  456 (92.1%)  275 (90.5%) |
| **ECL dataset**   - Controls - TBI TP1 - TBI TP2 | Plasma  Plasma  Plasma | Observations Observations  Observations | 121  231  209 | 19 (15.7%)  41 (17.7%)  47 (22.5%) | 102 (84.3%)  190 (82.3%)  162 (77.5%) |
| **ECL dataset**   - Controls - TBI TP1 - TBI TP2 | CSF  CSF  CSF | Observations  Observations Observations | 121  121  77 | 30 (24.8%)  20 (16.5%)  7 (9.1%) | 91 (75.2%)  101 (83.5%)  70 (90.9%) |
| Cross-platform (ECL vs PEA) | Plasma | Pairs | 485 | 103 (22.5%) | 355 (77.5%) |
| Cross-platform (ECL vs PEA) | CSF | Pairs | 253 | 81 (32%) | 172 (68%) |

**Suppl. Table 4. Sample inclusion and exclusions across datasets and analyses.**

For each dataset, compartment, and subgroup, counts are shown as initial observations, excluded observations (missing concentration values), and final included observations. Percentages are calculated within each row. "Observations" refer to single concentration values per analyte per sample (platform output). Exclusions reflect missing concentration values in the platform output. For cross-platform analyses, "pairs" refer to matched measurements where both platforms (electrochemiluminescence/ECL and Proximity Extension Assay/PEA) provided a non-missing concentration for the same participant × time point × compartment × analyte. In the cross-platform rows, "Excluded" reflects pairs that could not be formed because the concentration was missing on either platform for that matched measurement. Percentages are calculated within each row. Abbreviations: CSF:

Cerebrospinal fluid. TP1/2: Timepoint 1/2.

| **Mediators (platform used)** | **Early time point (day 1-3), time point 1** | **Late time point (day 4-8), time point 2** |
| --- | --- | --- |
| IL-6 (ECL)  IL-6 (PEA) | ↑ (Significantly higher than time point 2)  ↑ (Significantly higher than time point 2) | ↑  ↑ |
| IL-8 (ECL)  IL-8 (PEA) | ↑  ↑ | ↑  ↑ |
| IL-10 (ECL)  IL-10 (PEA) | ↑  ↑ | ↑  ↑ |
| TNF-α (ECL)  TNF-α (PEA) | ↑  → | ↑  ↑ |
| CSF1 (PEA) | ↑ | ↑ |
| CSF3 (PEA) | ↑ (Significantly higher than time point 2) | ↑ |
| IL-15 (PEA) | ↑ | ↑ |
| IL-17a (PEA) | ↑ | ↑ (Significantly higher than time point 1) |
| CCL7 (PEA) | ↑ | ↑ |
| CXCL12 (PEA) | ↑ | ↑ |
| IL-17c (PEA) | ↑ | ↑ |
| VEGFA (PEA) | → | ↑ |
| HGF (PEA) | ↑ | ↑ |
| OLR1 (PEA) | → | ↓ |
| TNFsf10 (PEA) | ↓ | → |
| TNFsf12 (PEA) | ↓ | ↓ |

**Suppl. Table 5. Temporal changes of inflammatory mediators in plasma – significant Mediators only.** Summary of mediator dynamics in plasma across the early (days 1–3) and late (days 4–8) post-injury time points, as measured by both electrochemiluminescence (ECL) and proximity extension assay (PEA). Comparisons between TBI patients and controls at each time point were performed using the Mann–Whitney U test, while paired comparisons within the TBI group were analyzed with the Wilcoxon signed-rank test. Arrows indicate the direction of change: ↑ = significantly increased compared to controls, ↓ = significantly decreased compared to controls, and → = no significant change. Where temporal differences between time points were significant, this is specified in parentheses**.**

| **Mediators (platform used)** | **Early time point (day 1-3), time point 1** | **Late time point (day 4-8), time point 2** |
| --- | --- | --- |
| IL-6 (ECL)  IL-6 (PEA) | ↑  ↑ | ↑  ↑ |
| IL-8 (ECL)  IL-8 (PEA) | ↑  ↑ | ↑  ↑ |
| IL-10 (ECL)  IL-10 (PEA) | ↑  ↑ | ↑  → |
| TNF-α (ECL)  TNF-α (PEA) | ↑  → | ↑  → |
| IL-2 (ECL)  IL-2 (PEA) | ↑  → | ↑  → |
| IL-4 (ECL)  IL-4 (PEA) | ↑  → | ↑  → |
| IFN-γ (ECL)  IFN-γ (PEA) | →  → | ↑  → |
| IL-1β (ECL)  IL-1β (PEA) | ↑  → | ↑  → |
| CCL11 (PEA) | ↑ | → |
| CCL13 (PEA) | ↑ | ↑ |
| CCL2 (PEA) | ↑ | ↑ |
| CCL3 (PEA) | ↑ | ↑ |
| CCL4 (PEA) | ↑ | ↑ |
| CCL7 (PEA) | ↑ | ↑ |
| CCL8 (PEA) | ↑ | ↑ |
| CXCL10 (PEA) | → | ↑ |
| IL-7 (PEA) | ↑ | ↑ |
| MMP1 (PEA) | ↑ | ↑ |
| OSM (PEA) | ↑ | ↑ |
| LTA (PEA) | ↓ | ↓ |
| TGFα (PEA) | ↓ | ↓ |
| TNFsf12 (PEA) | ↓ | ↓ |

**Suppl. Table 6. Temporal changes of inflammatory mediators in CSF - Significant Mediators only**. Summary of mediator dynamics in CSF across the early (days 1–3) and late (days 4–8) post-injury time points, as measured by both electrochemiluminescence (ECL) and proximity extension assay (PEA). Comparisons between TBI patients and controls at each time point were performed using the Mann–Whitney U test, while paired comparisons within the TBI group were analyzed with the Wilcoxon signed-rank test. Arrows indicate the direction of change: ↑ = significantly increased compared to controls, ↓ = significantly decreased compared to controls, and → = no significant change. Where temporal differences between time points were significant, this is specified in parentheses.

| **Mediator** | **Plasma change** | **CSF change** | **Platform** |
| --- | --- | --- | --- |
| IL-6 (ECL) | Increase | Increase | ECL |
| IL-6 (PEA) | Increase | Increase | PEA |
| IL-8 (ECL) | Increase | Increase | ECL |
| IL-8 (PEA) | Increase | Increase | PEA |
| IL-10 (ECL) | Increase | Increase | ECL |
| IL-10 (PEA) | Increase | Increase | PEA |
| TNF-α (ECL) | Increase | Increase | ECL |
| TNF-α (PEA) | Increase | No change | PEA |
| IL-2 | No change | Increase | ECL |
| IL-4 | No change | Increase | ECL |
| IFN-γ | No change | Increase | ECL |
| IL-1β | No change | Increase | ECL |
| CCL7 | Increase | Increase | PEA |
| TNFsf12 | Decrease | Decrease | PEA |
| CSF1 | Increase | No change | PEA |
| CSF3 | Increase | No change | PEA |
| IL-15 | Increase | No change | PEA |
| IL-17a | Increase | No change | PEA |
| CXCL12 | Increase | No change | PEA |
| IL-17c | Increase | No change | PEA |
| VEGFA | Increase | No change | PEA |
| HGF | Increase | No change | PEA |
| OLR1 | Decrease | No change | PEA |
| TNFsf10 | Decrease | No change | PEA |
| CCL11 | No change | Increase | PEA |
| CCL13 | No change | Increase | PEA |
| CCL2 | No change | Increase | PEA |
| CCL3 | No change | Increase | PEA |
| CCL4 | No change | Increase | PEA |
| CCL8 | No change | Increase | PEA |
| CXCL10 | No change | Increase | PEA |
| IL-7 | No change | Increase | PEA |
| MMP1 | No change | Increase | PEA |
| OSM | No change | Increase | PEA |
| LTA | No change | Decrease | PEA |
| TGFα | No change | Decrease | PEA |

**Supp Table 7. Overview of significantly altered mediators in plasma and CSF across platforms.** This table summarizes cytokine and chemokine alterations in TBI patients compared with controls across plasma and CSF, assessed using electrochemiluminescence (ECL) and proximity extension assay (PEA). Results are categorized as Increase, Decrease, or No change relative to controls. Only mediators with significant alterations in at least one compartment are included.

**Figures**

**
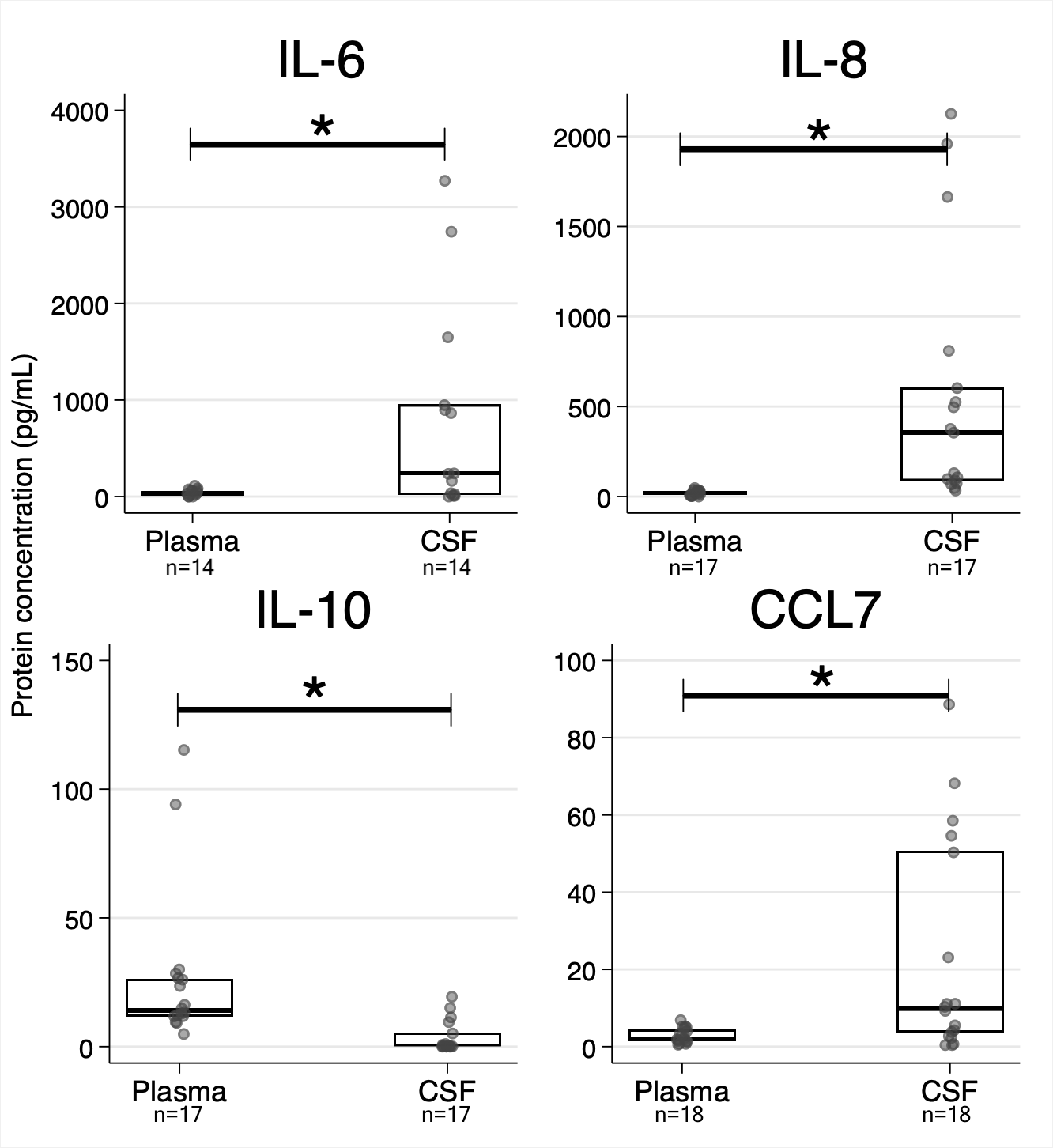
**

**Supp Figure 1. Significant compartment differences in mediators significantly elevated in both compartments in TBI patients using the PEA platform.** Boxplots compare paired plasma and CSF concentrations (pg/mL) of IL-6, IL-8, IL-10 and CCL7 measured by the PEA platform in TBI patients. The x-axis represents the fluid compartment, and the y-axis shows mediator concentrations in pg/mL. Boxplots show the median and interquartile range (25th–75th percentiles), with individual paired observations overlaid and sample size (n) for each compartment is indicated in each panel. Wilcoxon signed-rank tests revealed significantly higher concentrations of IL-6, IL-8 and CCL7 in CSF, while IL-10 was significantly higher in plasma. Significant differences after Benjamini–Hochberg FDR correction are indicated by * (q < 0.05).

**
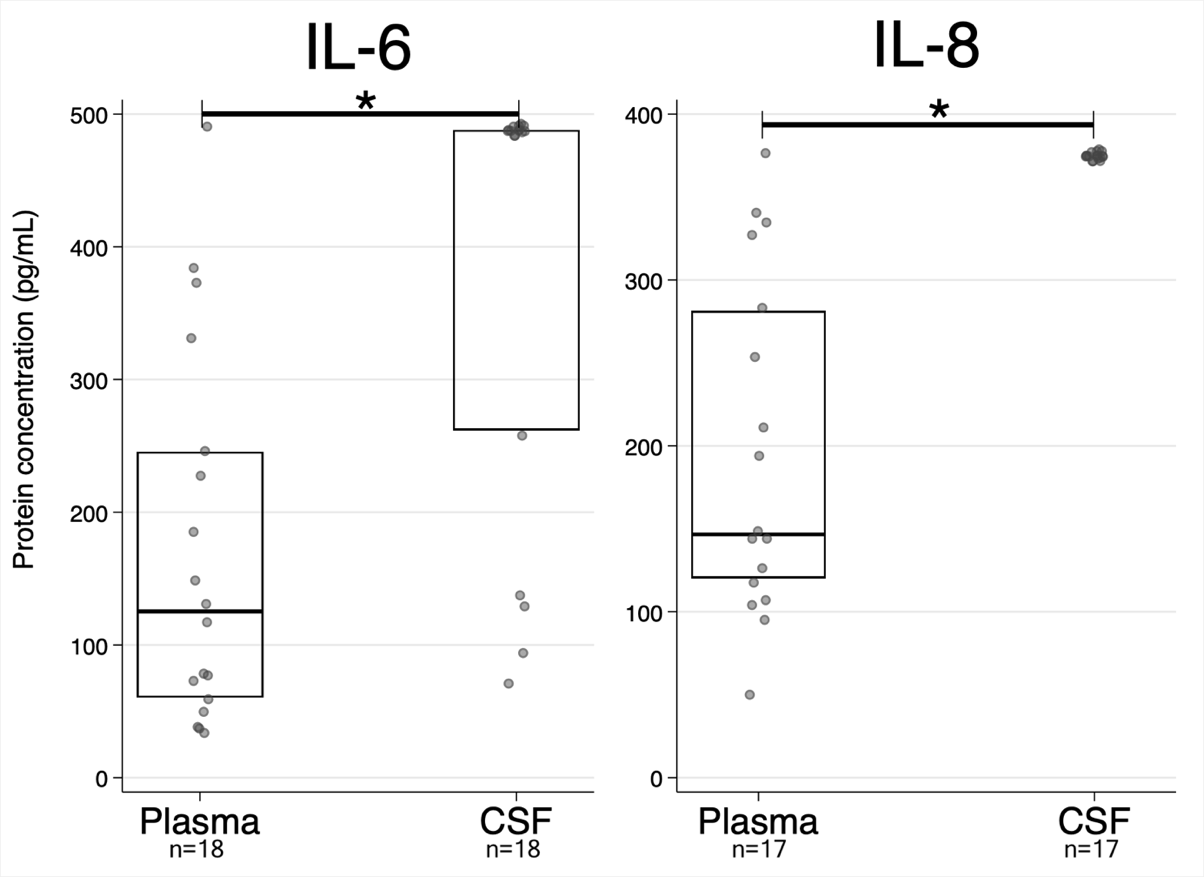
**

**Supp Figure 2. Significant compartment differences in mediators significantly elevated in both compartments in TBI patients using the ECL platform.** Boxplots compare paired plasma and CSF concentrations (pg/mL) of IL-6 and IL-8 measured by the electrochemiluminescence (ECL) platform in TBI patients. The x-axis represents the compartment fluid, and the y-axis indicates protein concentration. Boxplots show the median and interquartile range (25th–75th percentiles), with individual paired observations overlaid and sample size (n) for each compartment is indicated in each panel. Wilcoxon signed-rank tests revealed significantly higher concentrations of IL-6 and IL-8 levels in CSF compared to plasma. Notably, in IL-8, all CSF values reached the upper limit of quantification (ULOQ) and were therefore capped at this threshold. Significant differences after Benjamini–Hochberg FDR correction are indicated by * (q < 0.05).
